# Ti_4_O_7_/g-C_3_N_4_ for Visible Light Photocatalytic Oxidation of Hypophosphite: Effect of Mass Ratio of Ti_4_O_7_/g-C_3_N_4_

**DOI:** 10.3389/fchem.2018.00313

**Published:** 2018-07-24

**Authors:** Wei Guan, Zhenghua Zhang, Shichao Tian, Jianwei Du

**Affiliations:** ^1^South China Institute of Environmental Sciences, The Ministry of Environment Protection of PRC, Guangzhou, China; ^2^Graduate School at Shenzhen, Research Institute of Environmental Engineering and Nano-Technology, Tsinghua University, Shenzhen, China; ^3^Shenzhen Environmental Science and New Energy Technology Engineering Laboratory, Tsinghua-Berkeley Shenzhen Institute, Shenzhen, China

**Keywords:** photocatalysts, hypophosphite oxidation, graphitic carbon nitride, sub-stoichiometric titanium oxides, visible light irradiation

## Abstract

Hypophosphite wastewater treatment is still a critical issue in metallurgical processes and the oxidation of hypophosphite to phosphate followed by the precipitation of phosphate is an important strategy for hypophosphite wastewater treatment. Herein, Ti_4_O_7_/g-C_3_N_4_ photocatalysts with various mass ratios (Ti_4_O_7_ (m): g-C_3_N_4_ (m) = 0.5, 0.2, 0.1, and 0.05) were synthesized by a hydrolysis method and the effect of the mass ratio of Ti_4_O_7_ (m): g-C_3_N_4_ (m) on Ti_4_O_7_/g-C_3_N_4_ visible light photocatalytic oxidation of hypophosphite was evaluated. The as-prepared Ti_4_O_7_/g-C_3_N_4_ were characterized and confirmed by SEM, XPS, XRD and FTIR. Moreover, the specific surface area and the distribution of pore size of Ti_4_O_7_/g-C_3_N_4_ was also analyzed. Our results showed that Ti_4_O_7_/g-C_3_N_4_ exhibited remarkably improved photocatalytic performance on hypophosphite oxidation compared with g-C_3_N_4_ and meanwhile 1:2-Ti_4_O_7_/g-C_3_N_4_ with a mass ratio of 0.5 showed the best photocatalytic performance with the highest oxidation rate constant (17.7-fold and 91.0-fold higher than that of pure g-C_3_N_4_ and Ti_4_O_7_, respectively). The enhanced performance of photocatalytic oxidation of hypophosphite was ascribed to the heterojunction structure of Ti_4_O_7_/g-C_3_N_4_ with broader light absorption and significantly enhanced efficiency of the charge carrier (e^−^-h^+^) generation and separation. Additionally, the generated ·OH and ·${\text{O}}_{2}^{-}$ radicals contributed to the hypophosphite oxidation during the photocatalytic system.

## Introduction

Hypophosphite wastewater is produced in metallurgical processes where hypophosphite is a widely used reducing reagent for chemical nickel deposition (Gan et al., 2007; Huang et al., 2009). The discharge of hypophosphite wastewater may result in eutrophication and therefore the further treatment is required (Piveteau et al., 2017; Tian et al., 2017). Coagulants such as Fe have been widely used for phosphorus removal (Shih et al., 2013), however, the hypophosphite precipitants are not stable due to the high solubility constant (Zhao et al., 2017). As such, the pre-oxidation of hypophosphite to phosphate is very important for hypophosphite wastewater treatment so as to facilitate the following precipitation of phosphate in the form of insoluble salts precipitates.

Photocatalysis is considered to be a useful technology for water treatment with advantages of energy-free by using solar energy and high oxidation efficiency of pollutants by hydroxyl radicals (·OH) and superoxide radicals (·${\text{O}}_{2}^{-}$) generated during the photocatalytic process (Hao et al., 2017). The most commonly used TiO_2_ photocatalyst, however, is greatly limited in wide applications especially under visible light or sunlight due to its main drawback of wide band gap (3.2 eV) (Hao et al., 2016; Ma et al., 2018). Therefore, photocatalysts with wide range of response wavelength as well as good photogenerated charge separation properties are urgently required for photocatalytic applications.

Recently, graphite-like carbon nitride (g-C_3_N_4_) has attracted lots of attentions due to its advantages including small band gap of 2.73 eV, robust chemical stability over a wide pH range of 0–14 (Zhang et al., 2014; Li et al., 2016a,b). Nevertheless, limitations including low surface area and poor photogenerated charge separation properties still hinder the photocatalytic applications of g-C_3_N_4_ (Shao et al., 2017). Preparation of g-C_3_N_4_-based heterojunction is an alternative pathway to facilitate the enhancement of charge separation and photocatalytic performance (Masih et al., 2017). For instance, photocatalysts with *p*–*n* junction exhibited excellent photocatalytic performance in environmental and energy applications (Huang et al., 2015a,b, 2016; Zhang et al., 2018).

Magnéli phase titanium suboxides (Ti_n_O_2n−1_) are substoichiometric titanium oxides, where n is an integer between 4 and 10 (i.e., 4, 5, 6, and 8) (Zaky and Chaplin, 2014). Among various compositions of Ti_n_O_2n−1_, Ti_4_O_7_ has the properties of best conductivity (1,500 S cm^−1^) and the robust resistance to aggressive chemical conditions (Ganiyu et al., 2016). Construction of g-C_3_N_4_/Ti_4_O_7_ heterojunction significantly improved the photocatalytic performance as a result of the effective enhancement of charge separation was reported in our previous work (Guan et al., 2018). However, the effect of the mass ratio of Ti_4_O_7_ (m): g-C_3_N_4_ (m) of the g-C_3_N_4_/Ti_4_O_7_ heterojunction on photocatalytic performance is still unknown. In this study, the effect of the mass ratio of Ti_4_O_7_/g-C_3_N_4_ (Ti_4_O_7_ (m): g-C_3_N_4_ (m) = 0.5, 0.2, 0.1 and 0.05) on photocatalytic oxidation of hypophosphite was evaluated in view of the photocatalytic performance, optical and electrochemical properties as well as the contributions of ·OH and ·${\text{O}}_{2}^{-}$ radicals generated during the photocatalytic process. Our results indicated that Ti_4_O_7_/g-C_3_N_4_ with a mass ratio of 0.5 showed the highest rate constant of photocatalytic oxidation of hypophosphite (17.7-fold and 91.0-fold higher than that of pure g-C_3_N_4_ and Ti_4_O_7_, respectively).

## Materials and methods

### Chemicals

The reagents used for the preparation and performance characterization of g-C_3_N_4_/Ti_4_O_7_ were analytical grade and included sub-stoichiometric titanium oxide, melamine, urea, sodium hypophosphite, sodium sulfate, isopropanol, sodium hydroxide, sulfuric acid. All chemicals were bought from Sinopharm Chemical Reagent Co., Ltd. (Beijing, China). In addition, all solutions were prepared using freshly prepared Milli-Q water (Millipore, 18.2 MΩ cm).

### Preparation of Ti_4_O_7_/g-C_3_N_4_ photocatalysts

Graphite-like carbon nitride was prepared first using a liquid-based growth method (Sun et al., 2018). Then, g-C_3_N_4_ (2 g) and Ti_4_O_7_ (1, 0.4, 0.2, 0.1 g) were well mixed in the 0.1 mol/L NaOH solution (100 mL) using ultrasonication following by the annealing procedure at 160°C for 20 h. Subsequently, the as-prepared g-C_3_N_4_/Ti_4_O_7_ with various mass ratios (Ti_4_O_7_ (m): g-C_3_N_4_ (m) = 0.5, 0.2, 0.1, and 0.05) were dried at 60°C for 12 h before usage and noted as 1:2-Ti_4_O_7_/g-C_3_N_4_, 1:5-Ti_4_O_7_/g-C_3_N_4_, 1:10-Ti_4_O_7_/g-C_3_N_4_ and 1:20-Ti_4_O_7_/g-C_3_N_4_, respectively.

### Analysis and test methods

The as-prepared g-C_3_N_4_/Ti_4_O_7_ were characterized by scanning electron microscopy (SEM) (JEOL JSM-6701F), X-ray photoelectron spectroscopy (XPS) (Phi Quantern instrument with C 1s peak (284.8 eV) as the calibrated reference), X-ray diffraction (XRD) (model D/max RA, Rigaku Co., Japan), and fourier transforms infrared spectroscopy spectra (FTIR) (Bruker Tensor-27). The specific surface area and the pore size distribution of the as-prepared g-C_3_N_4_/Ti_4_O_7_ were calculated by Brunauer-Emmett-Teller (BET) equation and Barrett-Joyner-Halenda (BJH) method according to the N_2_ adsorption/desorption isotherms. The optical and electrochemical properties of the as-prepared g-C_3_N_4_/Ti_4_O_7_ were evaluated by Ultraviolet-visible diffraction spectra (UV-vis DRS) (UV-2450, Shimadzu, Japan) and CHI 660B electrochemical system in view of photocurrent (PC), cyclic voltammetry (CV) and electrochemical impedance spectroscopy (EIS). The contributions of ·OH and ·${\text{O}}_{2}^{-}$ radicals generated during the visible light photocatalytic process were identified by comparing the efficiencies of hypophosphite oxidation in the absence and presence of isopropanol (IPA) and N_2_ purging.

### Analysis of photocatalytic performance

The photocatalytic performance of g-C_3_N_4_/Ti_4_O_7_ was characterized by photocatalytic oxidation of hypophosphite with the concentration of hypophosphite measured by ion chromatography using a 732 IC detector. A metal-halide lamp (35 W, Philips) with the light strength of ~5 mW cm^−2^ was used as the light source and a UV-cutoff filter of 420 nm was used to provide the visible light with the wavelength over 420 nm. Before the photocatalytic experiments, g-C_3_N_4_/Ti_4_O_7_ with various mass ratios (10 mg) dispersed in 100 mg L^−1^ hypophosphite aqueous solution (100 mL) were continuously stirred for 30 min in the dark to achieve the adsorption–desorption equilibrium. After that, the visible light photocatalytic oxidation of hypophosphite was conducted with the above solutions exposing to the visible light irradiation and sampling conducted at 1 h intervals over 6 h experimental period. More detailed information about the photocatalytic experiment can be found in our most recent paper (Guan et al., 2018). The oxidation efficiency of hypophosphite (η) was calculated using the following equation (Guan et al., 2017):

(1) $$\eta =\frac{{\text{C}}_{\text{0}}\;-\;{\text{C}}_{\text{t}}}{{\text{C}}_{\text{0}}}\;\times \text{100}\%$$

where C_0_ and C_t_ represent the concentrations of hypophosphite at initial and given time, respectively.

## Results and discussion

### Materials characterization

**Figure 1:**
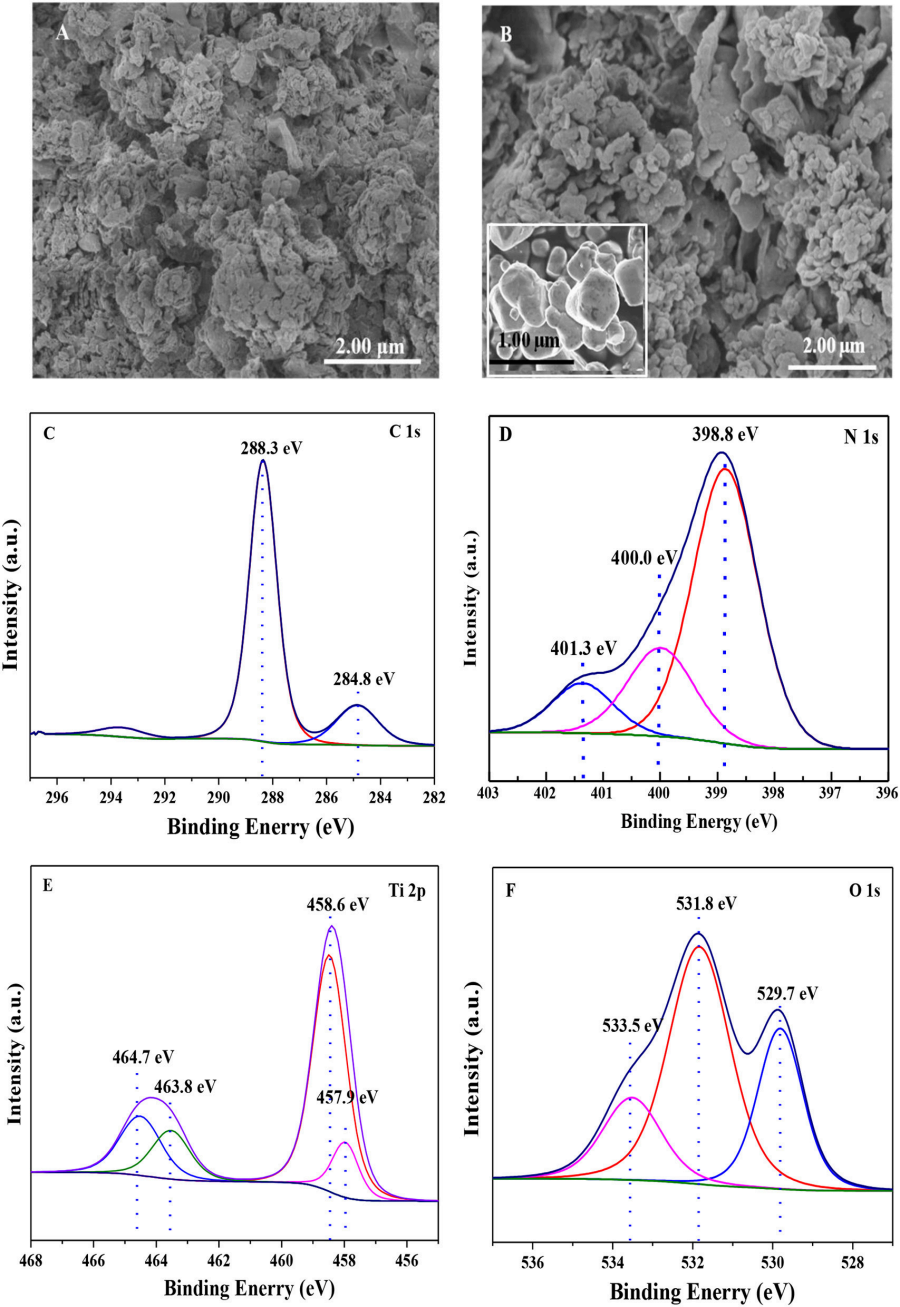
The SEM images of **(A)** g-C_3_N_4_, **(B)** Ti_4_O_7_/g-C_3_N_4_ photocatalysts; and XPS spectrum of **(C)** C 1s, **(D)** N 1s, **(E)** Ti 2p, **(F)** O 1s of Ti_4_O_7_/g-C_3_N_4_ photocatalyst.

The SEM images of pure g-C_3_N_4_ and Ti_4_O_7_/g-C_3_N_4_ photocatalysts were shown in Figure 1 that g-C_3_N_4_ had a sheet-like structure (Figure 1A) and spheroidal Ti_4_O_7_ crystals were deposited on the surface of C_3_N_4_ (Figure 1B)_._ The corresponding XPS high resolution spectra of Ti_4_O_7_/g-C_3_N_4_ were analyzed as shown in Figure 1. There were two components for the XPS spectra of C 1s core level (Figure 1C), the standard reference carbon (284.8 eV) and the sp^2^ bonded C in N=C(–N)_2_ (288.3 eV) (Jo and Natarajan, 2015). With regard to the N 1s spectra, there were three peaks (Figure 1D), the main peak at 398.8 eV assigned to sp^2^ nitrogen (C=N–C) involved in triazine rings, the peak at 400.0 eV originated from the tertiary nitrogen bonded to carbon atoms in the form of N–(C)_3_ (Wu et al., 2013) and the peak at 401.3 eV ascribed to amino functions (C–N–H) (Gao et al., 2014). These assignments of C 1s and N 1s were agreed well with the XPS results of g-C_3_N_4_ reported previously. Meanwhile, Ti_4_O_7_ is a mixed-valence compound with two evenly occupied Ti^4+^ (3d^0^) and Ti^3+^ (3d^1^) configurations. Four peaks were observed for Ti 2p spectra (Figure 1E) that two broad peaks at 458.6 eV and 464.7 eV were respectively assigned to Ti 2p_1/2_ and Ti 2p_3/2_ peaks of Ti^4+^, and another two peaks at 457.9 eV and 463.8 eV were assigned to Ti^3+^ (Zeng et al., 2017). In terms of the O 1s spectra, three peaks were observed (Figure 1F) that the peak at 533.5 eV was assigned to the C–O functional groups, and the peaks at 531.8 and 529.7 eV were ascribed to the OH–Ti and O–Ti bonds (Li et al., 2017). The XPS results confirmed the presence of Ti_4_O_7_ on the surface of g-C_3_N_4_ with covalent bonds.

**Figure 2:**
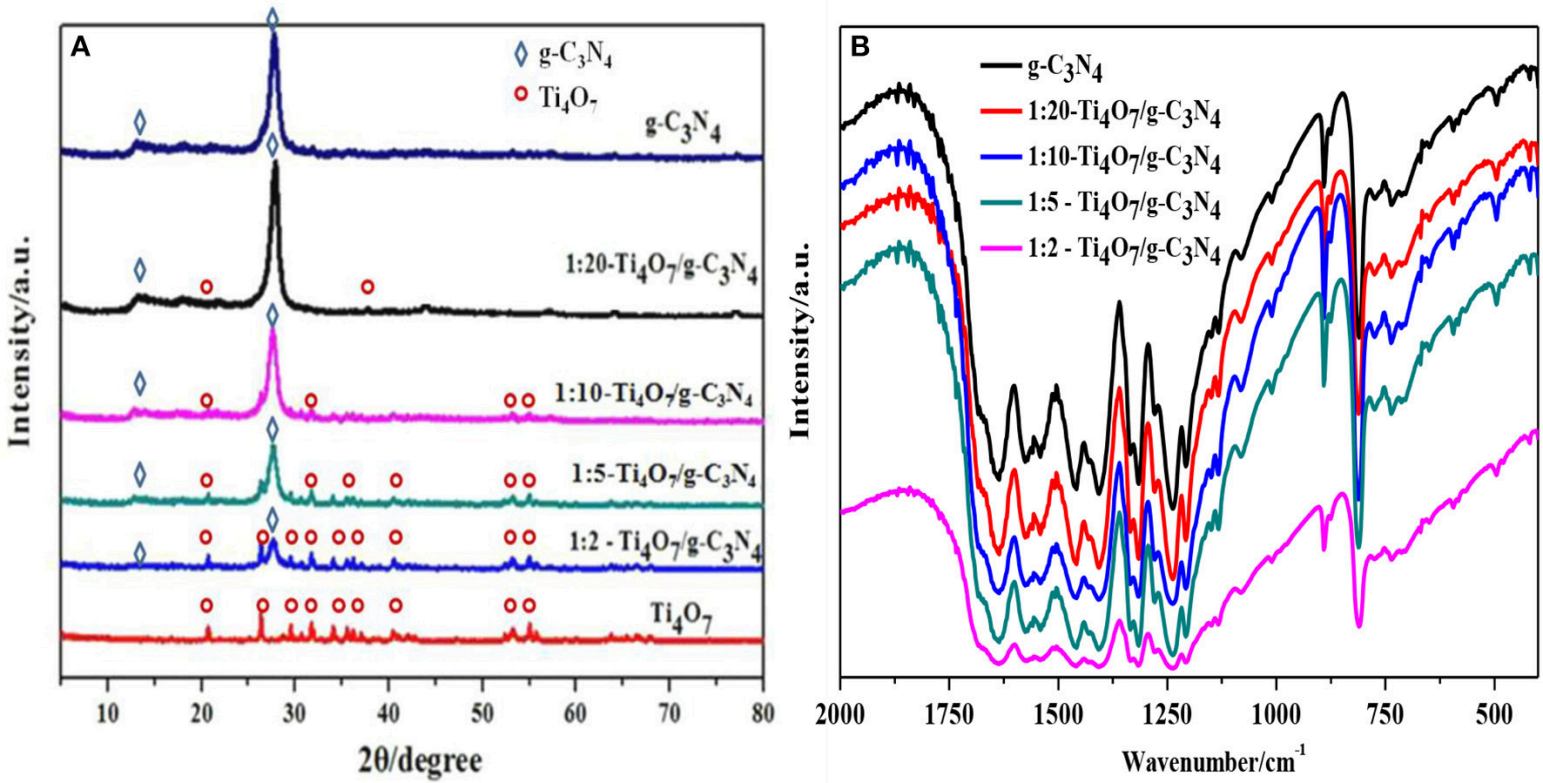
Structure and morphology analysis of g-C_3_N_4_ and Ti_4_O_7_/g-C_3_N_4_ photocatalysts: **(A)** XRD patterns analysis; **(B)** FTIR spectrometer analysis.

The XRD phase structures of Ti_4_O_7_/g-C_3_N_4_ photocatalysts with various mass ratios were shown in Figure 2A. Peaks at 13.10° and 27.40° were indexed as (1 0 0) plane of tri-s-triazine units and (0 0 2) plane of the conjugated aromatic system of g-C_3_N_4_, respectively (Liang and Zhu, 2016). Meanwhile, major peaks of Ti_4_O_7_ including 20.7, 26.3, 29.5, 31.7, 34.0, 36.3, 40.5, 53.1, 55.0, 63.8, and 66.4° were also found (Guo et al., 2016). With increase of the mass ratio of Ti_4_O_7_ (m): g-C_3_N_4_ (m), the intensity of the Ti_4_O_7_ peaks became stronger while that of the C_3_N_4_ peaks became weaker. Furthermore, Ti_4_O_7_/g-C_3_N_4_ especially 1:2-Ti_4_O_7_/g-C_3_N_4_ (mass ratio of Ti_4_O_7_/g-C_3_N_4_ of 0.5) matched well with the reference of pure Ti_4_O_7_ and g-C_3_N_4_, indicating that the main structures of Ti_4_O_7_ and g-C_3_N_4_ were not destroyed during the synthesis process of Ti_4_O_7_/g-C_3_N_4_.

The as-prepared Ti_4_O_7_/g-C_3_N_4_ photocatalysts with various mass ratios were further characterized by FTIR as shown in Figure 2B. Typical absorption peaks of 1,230–1,630 cm^−1^ were attributed to tri-s-triazine ring moieties of g-C_3_N_4_. For example, the absorption peaks of 1,638 and 1,570 cm^−1^ were related to C = N stretching, and the peaks of 1,474, 1,410, 1,322, 1,241 cm^−1^ were attributed to C–N stretching. Meanwhile, the sharp peak of 810 cm^−1^ was due to the bending vibration of heptazine rings, indicating that the heptazine units might exist in C_3_N_4_ (Hatamie et al., 2018). With increase of the mass ratio of Ti_4_O_7_/g-C_3_N_4_, the absorption intensity related to the vibrational bands of g-C_3_N_4_ became weaker. The presence of Ti_4_O_7_ in Ti_4_O_7_/g-C_3_N_4_ restricted the evolution trend of g-C_3_N_4_ and thus alleviated the severe stacking of aromatic units of g-C_3_N_4_. Additionally, the delocalized π-π conjugated electronic system of g-C_3_N_4_ facilitated the transfer of photogenerated electron-hole pairs during the photocatalytic process (Jourshabani et al., 2018).

**Figure 3:**
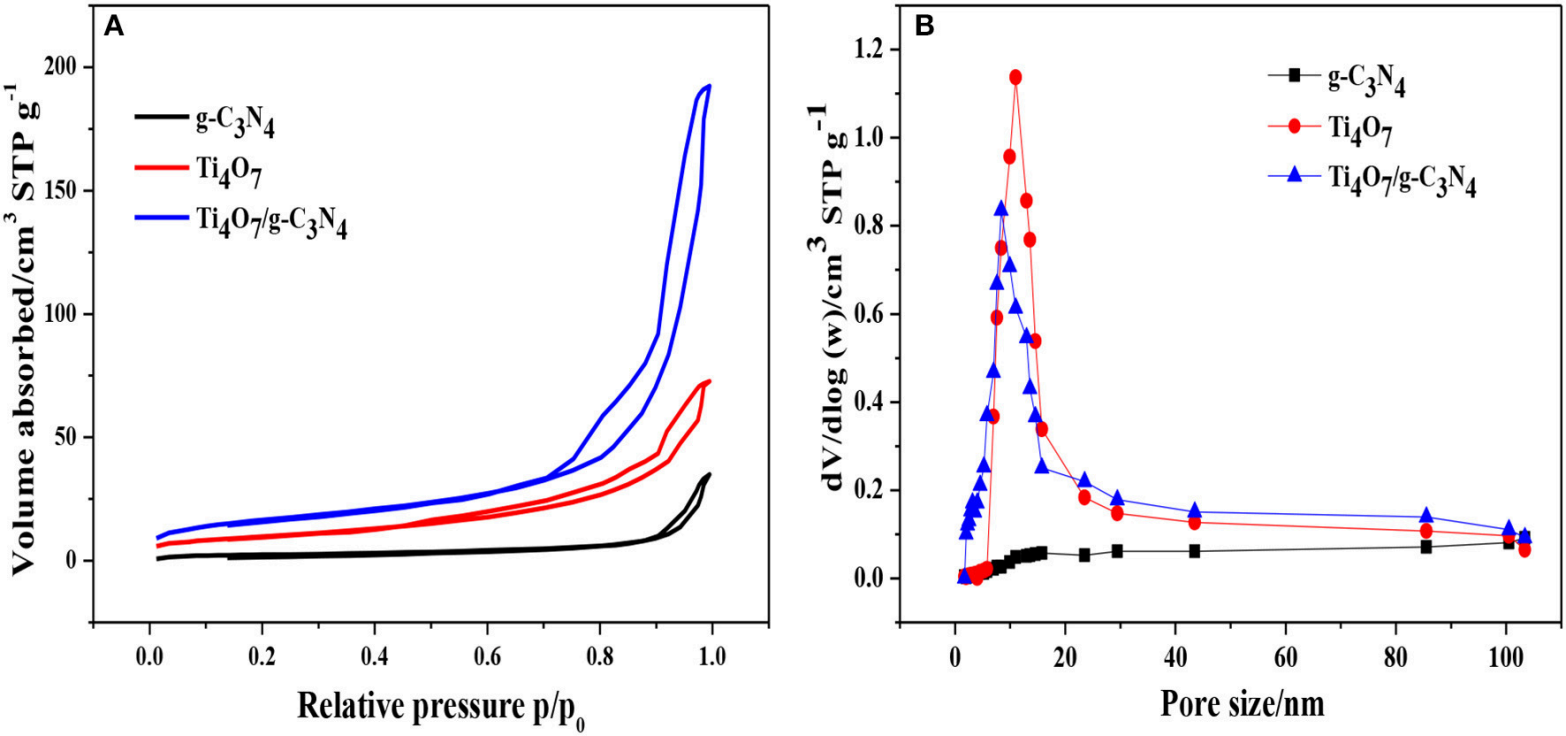
**(A)** N_2_ adsorption-desorption isotherms and **(B)** pore size distribution of g-C_3_N_4_, pure Ti_4_O_7_ and 1:2-Ti_4_O_7_/g-C_3_N_4_ photocatalysts.

The nitrogen adsorption-desorption isotherms and pore size distribution of pure Ti_4_O_7_, g-C_3_N_4_ and 1:2-Ti_4_O_7_/g-C_3_N_4_ photocatalysts were shown in Figure 3. The specific surface area calculated according to Figure 3A was 116.0, 56.5, and 174.0 m^2^·g^−1^ for Ti_4_O_7_, g-C_3_N_4_ and 1:2-Ti_4_O_7_/g-C_3_N_4_, respectively. Generally, a catalyst with larger surface area could offer more active sites for adsorption and photodegradation toward organic pollutants, resulting in an enhanced photodecomposition activity (Dong et al., 2015). As such, compared with pure Ti_4_O_7_ and g-C_3_N_4_, 1:2-Ti_4_O_7_/g-C_3_N_4_ with larger specific surface area would facilitate photocatalytic oxidation of hypophosphite. Moreover, the pore size distribution of 1:2-Ti_4_O_7_/g-C_3_N_4_ was between 5 and 15 nm and that of pure Ti_4_O_7_ was mainly in the range of 10–25 nm, while no obvious mesopores were observed for g-C_3_N_4_ (Figure 3B).

### Photocatalytic performance analysis

**Figure 4:**
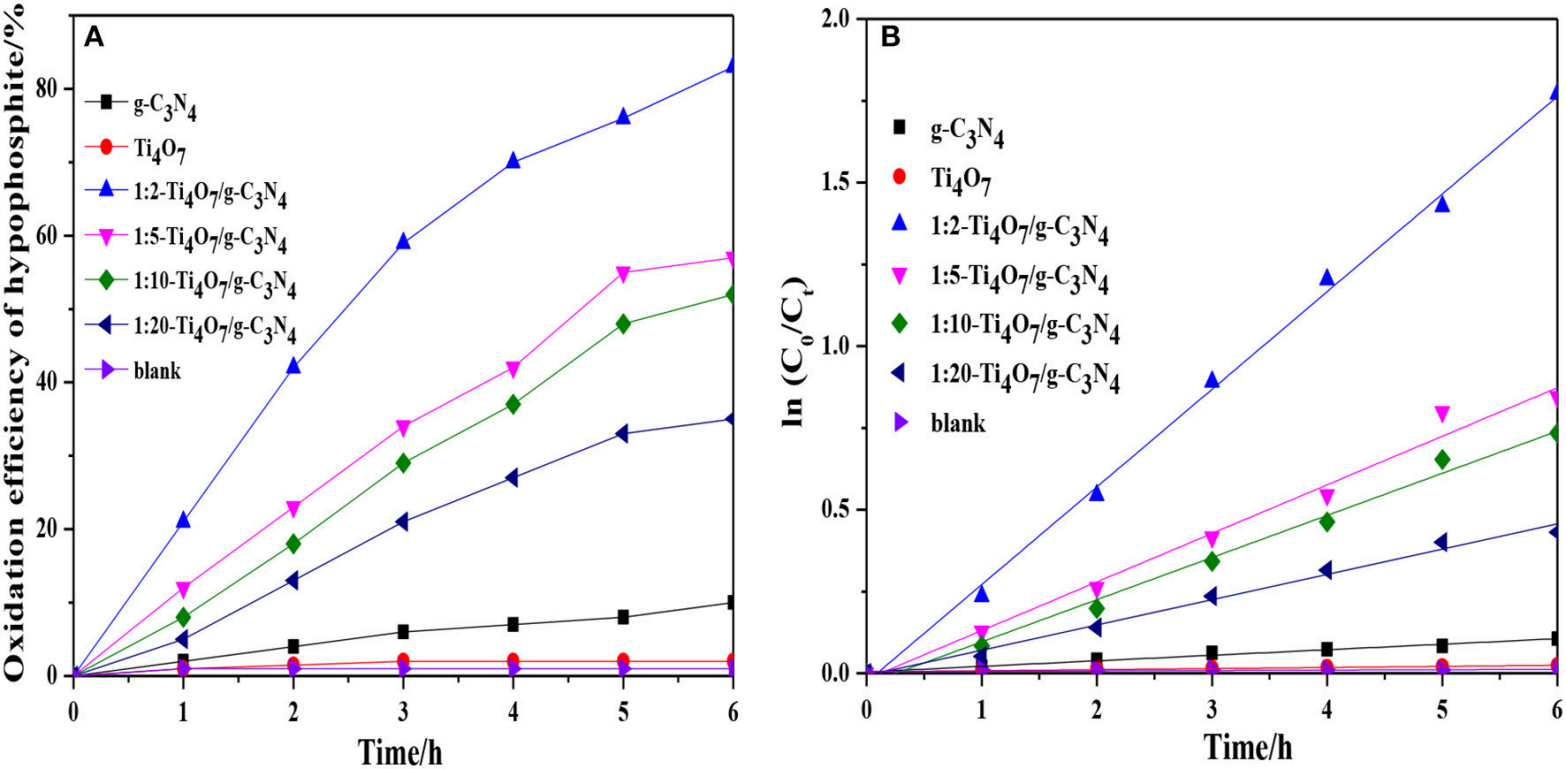
**(A)** The oxidation efficiency of hypophosphite for different photocatalysts; **(B)** Plot of ln(C_0_/C_t_) against reaction time for the catalytic oxidation of hypophosphite using different photocatalysts.

The photocatalytic performance of different photocatalysts was analyzed and compared as shown in Figure 4A. Ti_4_O_7_/g-C_3_N_4_ exhibited the significantly improved efficiency in hypophosphite oxidation under visible light irradiation (λ > 420 nm) in comparison to the pure g-C_3_N_4_ and Ti_4_O_7_. Additionally, the oxidation efficiency of hypophosphite was increased with increase of the mass ratio of Ti_4_O_7_/g-C_3_N_4_ and 1:2-Ti_4_O_7_/g-C_3_N_4_ illustrated the best photocatalytic performance with the highest oxidation efficiency of 83.6%. Furthermore, the photocatalytic oxidation reaction of hypophosphite was well fitted by pseudo first-order kinetic (*R*^2^ > 0.95) as shown in Figure 4B and the rate constant of 1:2-Ti_4_O_7_/g-C_3_N_4_ was 17.7-fold and 91.0-fold higher than that of pure g-C_3_N_4_ and Ti_4_O_7_, respectively. The low efficiency of photocatalytic oxidation of hypophosphite for pure g-C_3_N_4_ was possibly because of the rapid combination of electron-hole pairs (Yan et al., 2018). Similarly, the photocatalytic performance of pure Ti_4_O_7_ was extremely limited herein, which was mainly due to the wide band gap of 2.9 eV of pure Ti_4_O_7_ (Maragatha et al., 2017). Nevertheless, the efficiency of photocatalytic oxidation of hypophosphite was remarkably improved especially for 1:2-Ti_4_O_7_/g-C_3_N_4_ possibly as a result of the Ti_4_O_7_/g-C_3_N_4_ heterojunction with effectively increased recombination lifetime of electron-hole pairs and better charge transfer during the photocatalytic process, which was discussed in the following parts.

### Optical properties analysis

**Figure 5:**
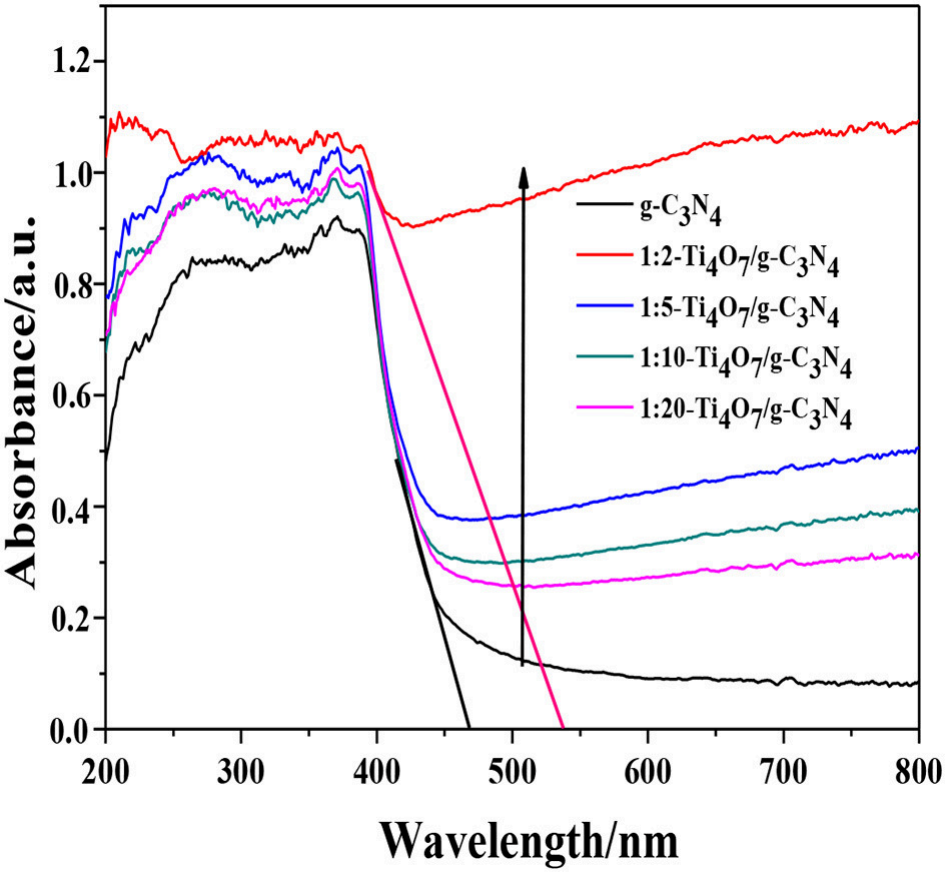
UV–vis DRS spectra of different photocatalysts.

Generally, the band gap of a semiconductor is related to its photocatalytic performance, because it determines the absorption properties of the incident photon, the recombination lifetime of the electron-hole pairs and transfer of charge carriers. As shown in Figure 5, Ti_4_O_7_/g-C_3_N_4_ showed a distinct red-shift in comparison to pure g-C_3_N_4_ in the UV–vis diffuse reflectance spectra. Moreover, the absorption intensities ranging from 450 to 800 nm gradually strengthened with increase of the mass ratio of Ti_4_O_7_ (m): g-C_3_N_4_ (m), indicating that the Ti_4_O_7_/g-C_3_N_4_ heterojunction with good interaction between Ti_4_O_7_ and g-C_3_N_4_ facilitated the enhancement of visible-light harvesting (Chang et al., 2018). The band gap of Ti_4_O_7_/g-C_3_N_4_ was determined such that (Ai et al., 2018):

(2) $${\left(\alpha \text{h}\nu \right)}^{2}=A(\text{h}\nu -{\text{E}}_{\text{g}})$$

where α is the optical absorption coefficient; h is the Plank's constant; ν is the photonic frequency; A is the proportionality constant; E_g_ is the band gap.

The band gap followed the order: 1:2-Ti_4_O_7_/g-C_3_N_4_ (2.07 eV) < 1:5-Ti_4_O_7_/g-C_3_N_4_ (2.25 eV) < 1:10-Ti_4_O_7_/g-C_3_N_4_ (2.33 eV) < 1:20-Ti_4_O_7_/g-C_3_N_4_ (2.43 eV) < pure g-C_3_N_4_ (2.70 eV). As such, the narrowed band gap of Ti_4_O_7_/g-C_3_N_4_ would effectively enhance the photoabsorption efficiency, which thus contributed to the improved efficiency of photocatalytic oxidation of hypophosphite as shown in Figure 4.

### Electrochemical properties analysis

**Figure 6:**
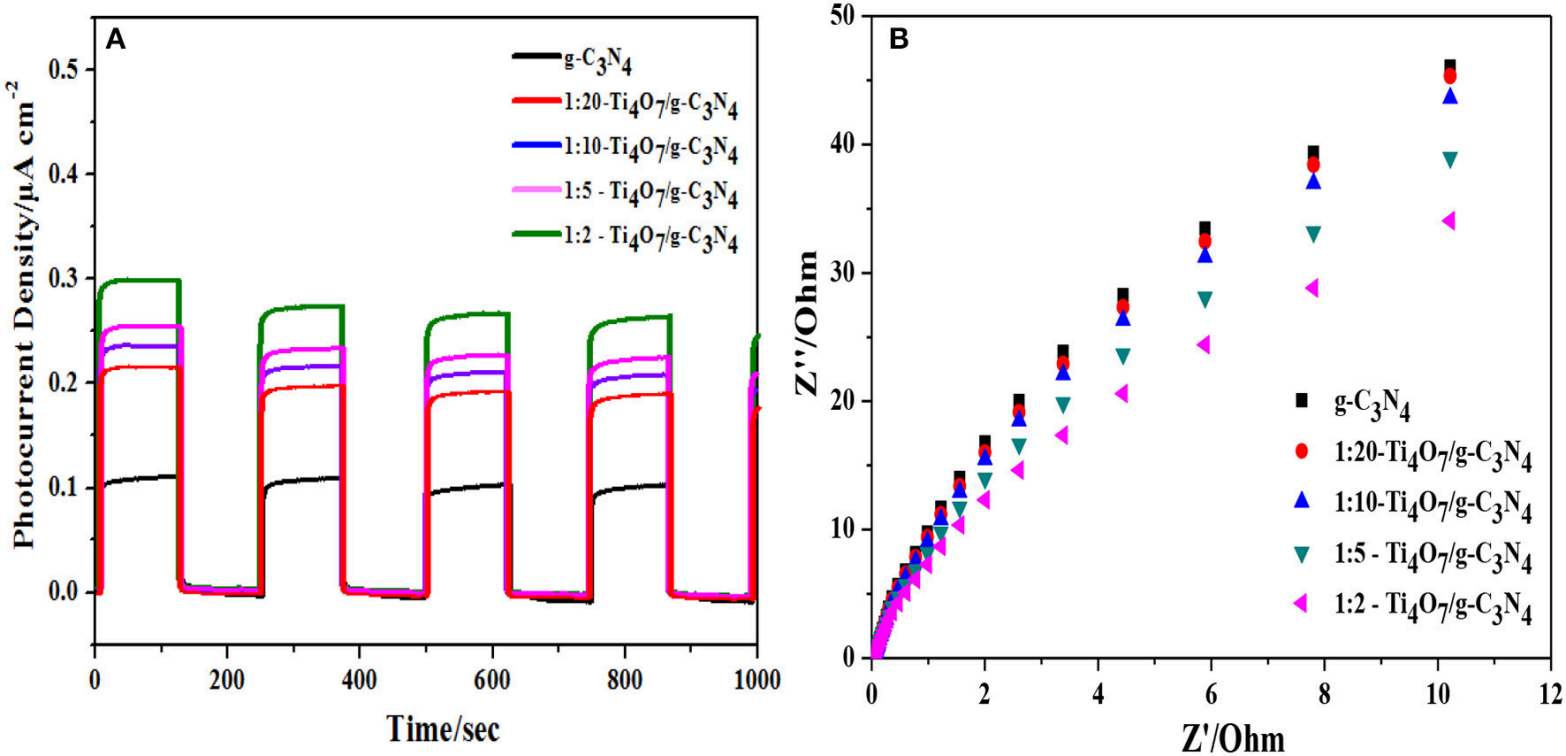
Electrochemical properties analysis: **(A)** photocurrent density and **(B)** EIS Nyquist plots of different photocatalysts.

CV and EIS results can indicate the combination efficiency of electron-hole pairs during the photocatalytic process. As shown in Figure 6A, the transient photoelectrochemical response current density of Ti_4_O_7_/g-C_3_N_4_ increased with the increase of the mass ratio of Ti_4_O_7_/g-C_3_N_4_. 1:2-Ti_4_O_7_/g-C_3_N_4_ had the highest photocurrent density of 0.30 μA cm^−2^, while the photocurrent density for pure g-C_3_N_4_ was only 0.10 μA cm^−2^. The enlarged photocurrent density of Ti_4_O_7_/g-C_3_N_4_ suggested the more efficient charge carrier (e^−^-h^+^) generation on the Ti_4_O_7_/g-C_3_N_4_ surface (Liu et al., 2017). Herein, the electrons in the valence band of g-C_3_N_4_ were excited upon visible light irradiation and then migrated to the conduction band of Ti_4_O_7_ with effectively enhanced efficiency of the charge carrier (e^−^-h^+^) generation and separation (Samanta and Srivastava, 2017), which improved the photocatalytic performance as indicated in Figure 4. In addition, the photoelectrochemical response current density of Ti_4_O_7_/g-C_3_N_4_ switched reversibly and was unchanged after repetitive ON/OFF illumination cycles, indicating the good photoelectrochemical stability of Ti_4_O_7_/g-C_3_N_4_.

Charge transfer of photocatalysts is another very important factor determining the photocatalytic performance (Kang et al., 2016). EIS was applied to analyze the photogenerated electron transfer process of Ti_4_O_7_/g-C_3_N_4_. As shown in Figure 6B, the arc radius decreased gradually with increase of the mass ratio of Ti_4_O_7_/g-C_3_N_4_ and the arc radius was lowest for 1:2-Ti_4_O_7_/g-C_3_N_4_. It is well known that the radius of Nyquist circle is related to the interfacial charge transfer that the smaller radius of Nyquist circle indicates a faster interfacial charge transfer and lower recombination rate of electron and hole (Guo et al., 2017). Herein, the decreased arc radius of Ti_4_O_7_/g-C_3_N_4_ indicated that 1:2-Ti_4_O_7_/g-C_3_N_4_ in comparison to pure g-C_3_N_4_ would also facilitate the photocatalytic oxidation of hypophosphite as evident in Figure 4.

### Proposed mechanism

**Figure 7:**
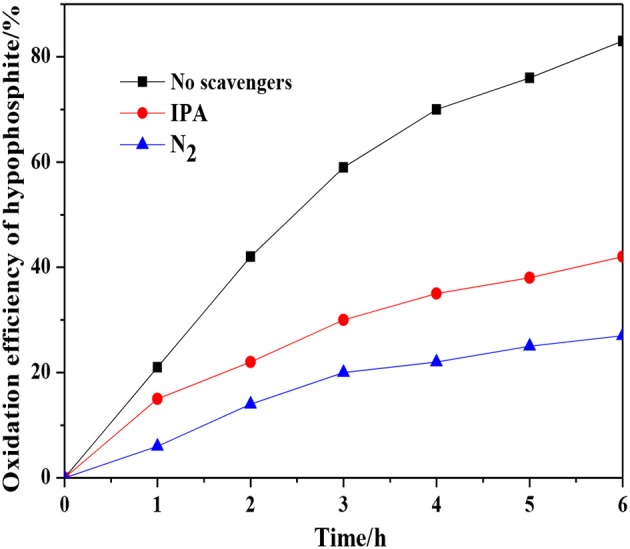
Effect of radical scavengers on the photocatalytic oxidation of hypophosphite by the 1:2-Ti_4_O_7_/g-C_3_N_4_ photocatalyst.

Reactive oxygen species (ROS) including ·${\text{O}}_{2}^{-}$ and ·OH would be generated during the photocatalytic system (Huang et al., 2017a,b). Herein, the contributions of ·OH and ·${\text{O}}_{2}^{-}$ to the photocatalytic oxidation of hypophosphite were analyzed by evaluation of the photocatalytic oxidation efficiency of hypophosphite in the presence of isopropanol (IPA) acting as the scavenger of ·OH (Tian et al., 2015) and N_2_ purging applied to reduce the superoxide ·${\text{O}}_{2}^{-}$ radicals (Zhang et al., 2017). As shown in Figure 7, the oxidation efficiency of hypophosphite was decreased to 42 and 27% in the presence of IPA and N_2_, respectively. In contrast, that value was 83% without radical scavengers. These results indicated that ·OH and ·${\text{O}}_{2}^{-}$ radicals significantly contributed to the photocatalytic oxidation of hypophosphite and other radicals such as singlet oxygen (^1^O_2_) and peroxyl (RO_2_·) may also make a contribution as evident by the lowest efficiency of 27% rather than 0% (Huang et al., 2017a).

**Figure 8:**
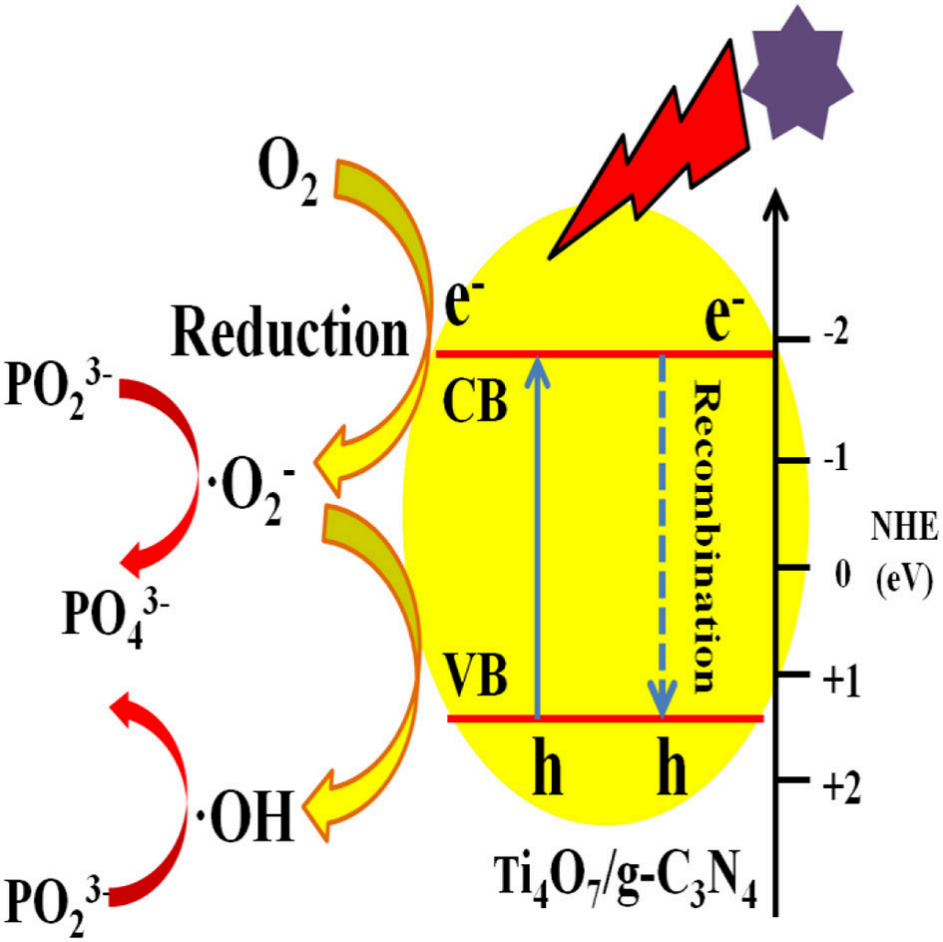
Schematic of the mechanism of photocatalytic oxidation of hypophosphite by Ti_4_O_7_/g-C_3_N_4_ photocatalysts.

The photocatalytic mechanism of Ti_4_O_7_/g-C_3_N_4_ was proposed as shown in Figure 8. Under visible light irradiation, electrons in the valence band of g-C_3_N_4_ were excited and then the excited electrons migrated to the conduction band of Ti_4_O_7_ through the heterojunction surface of Ti_4_O_7_/g-C_3_N_4_ with the generation of holes in the valence band of g-C_3_N_4_ (Zhu et al., 2018). The electrons on the surface of Ti_4_O_7_ could easily react with O_2_ adsorbed on the Ti_4_O_7_/g-C_3_N_4_ surface to generate ·${\text{O}}_{2}^{-}$ radicals for hypophosphite oxidation. Meanwhile, the active ·OH radicals with high redox potential were generated through ·${\text{O}}_{2}^{-}$ radicals, which further facilitated the oxidation of hypophosphite (Nosaka and Nosaka, 2017; Guan et al., 2018). Therefore, it can be concluded that the oxidation of hypophosphite was mainly ascribed to ·OH and ·${\text{O}}_{2}^{-}$ radicals generated during the photocatalytic process as shown in Figure 8.

### Analysis of stability

**Figure 9:**
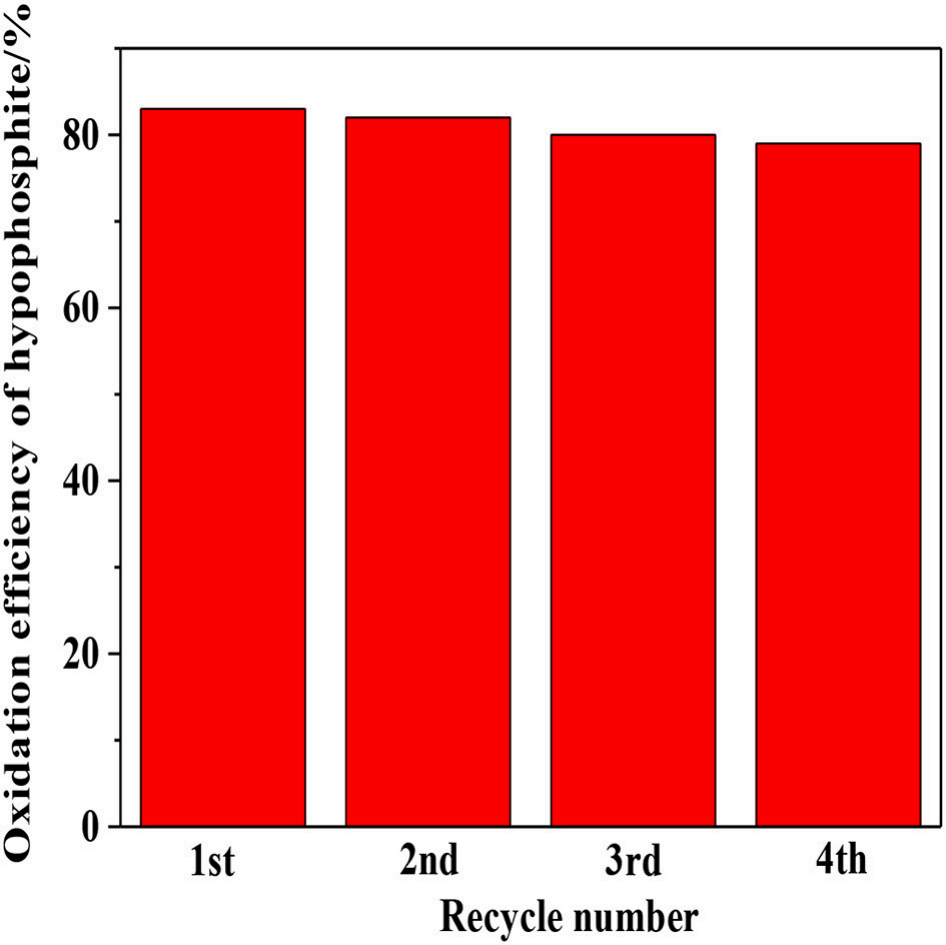
The stability analysis of the 1:2-Ti_4_O_7_/g-C_3_N_4_ photocatalyst.

The stability of photocatalyst is another vital consideration to evaluate the photocatalytic performance. As shown in Figure 9, relatively robust reusability was exhibited by 1:2-Ti_4_O_7_/g-C_3_N_4_ photocatalyst that the efficiency of photocatalytic oxidation of hypophosphite was almost stable in the range of 79–84% after four repetitive experiments. The slight decrease of oxidation efficiency was possibly due to the inevitable mass loss of photocatalyst during the recycling process.

## Conclusion

In this work, Ti_4_O_7_/g-C_3_N_4_ with various mass ratios (Ti_4_O_7_ (m): g-C_3_N_4_ (m) = 0.5, 0.2, 0.1, and 0.05) were synthesized by a hydrolysis method and the effect of the mass ratio of Ti_4_O_7_/g-C_3_N_4_ on Ti_4_O_7_/g-C_3_N_4_ visible light photocatalytic oxidation of hypophosphite was evaluated. Ti_4_O_7_/g-C_3_N_4_ exhibited remarkably improved photocatalytic performance on hypophosphite oxidation in comparison to pure g-C_3_N_4_ and 1:2-Ti_4_O_7_/g-C_3_N_4_ with a mass ratio of 0.5 showed the best photocatalytic performance with the highest oxidation rate constant of photocatalytic (17.7-fold and 91.0-fold higher than that of pure g-C_3_N_4_ and Ti_4_O_7_, respectively). The heterojunction structure of Ti_4_O_7_/g-C_3_N_4_ with broader light absorption significantly enhanced the efficiency of the charge carrier (e^−^-h^+^) generation and separation. ·OH and ·${\text{O}}_{2}^{-}$ radicals generated during the photocatalytic process were the main radicals contributing to the oxidation of hypophosphite.

## Author contributions

WG: experiment. ZZ: paper writing. ST: data analysis. JD: sample analysis.

### Conflict of interest statement

The authors declare that the research was conducted in the absence of any commercial or financial relationships that could be construed as a potential conflict of interest.
